# The N-Terminal Domain of EspF Induces Host Cell Apoptosis after Infection with Enterohaemorrhagic *Escherichia coli* O157:H7

**DOI:** 10.1371/journal.pone.0055164

**Published:** 2013-01-25

**Authors:** Suhui Zhao, Ying Zhou, Chunhui Wang, Yu Yang, Xianbo Wu, Yao Wei, Li Zhu, Wei Zhao, Qiwei Zhang, Chengsong Wan

**Affiliations:** Department of Microbiology, School of Public Health and Tropical Medicine, Southern Medical University, Guangzhou, People’s Republic of China; Beijing Institute of Microbiology and Epidemiology, China

## Abstract

Enterohemorrhagic *Escherichia coli* (EHEC) employs a type III secretion system (TTSS) to export the translocator and effector proteins required for mucosal colonization. As an important bacterial effector protein in locus of enterocyte effacement four, the EspF protein causes F-actin filament aggregations to form attaching and effacing (A/E) lesions, and induces the destruction of brush-border microvilli and cytoskeletal rearrangements to form pedestals. However, the molecular pathogenesis of A/E lesions due to EHEC O157:H7 infection is unclear. In this study, we constructed an *espF*-deficient mutant (Δ*espF*) with a 162-bp deletion in the N-terminal domain by using overlap extension PCR. The results showed that EHEC EspF translocated into intestinal epithelial cells, targeted mitochondria and induced apoptosis. The Δ*espF* mutant, compared to EHEC prototype Guangzhou strain, had lower cell attachment and effacement abilities, lower caspase-9/3 and lactate dehydrogenase levels, lower bacterial adhesion, weaker mitochondria apoptosis, and a higher mouse survival rate. Our results demonstrate the probable function of the EspF N-terminal domain, which targets mitochondria and binds mitochondria heat shock protein 70 to induce cell apoptosis via A/E lesions. These findings may be invaluable in clarifying the molecular pathogenesis of EspF of EHEC O157:H7.

## Introduction

Enterohemorrhagic *Escherichia coli* O157:H7 are important food-borne causative agents that cause sporadic outbreaks of illness such as diarrhea, hemorrhagic colitis (HC), haemolytic uraemic syndrome (HUS) and thrombotic thromobocytopenic porpura (TTP) [1], [2], [3]. The outbreaks and dissemination of EHEC O157:H7 persist, posing a great threat to human health and posing a global public health challenge.

EHEC employs a type III secretion system (TTSS) to export the translocator and effector proteins required for mucosal colonization [4]. The TTSS is encoded in a pathogenicity island called the locus of enterocyte effacement (LEE, 35–43 kb), consisting of LEE1 - LEE5 and several transcriptional units, encoded a type III secretion system (TTSS) which exports the translocator and effector proteins responsible for mucosal colonization [5]. EHEC O157:H7 adheres to the brush border of epithelial cells of the host’s large intestine; subsequently, TTSS translocates effectors such as Tir, Map, EspG, EspF, and EspH into host cells. Such translocation results in F-actin filaments aggregating to form attaching and effacing (A/E) intestinal lesions [6], [7], inducing the destruction of brush-border microvilli and cytoskeletal rearrangements to form pedestals [8].

EspF is an intrinsically disordered protein (IDP) that contains a transiently α-helical N-terminus and dynamic C-terminus [9]. The *espF* gene (747 bp, GenBank ID: 960871) locates between nt 4,658,240 to 4,658,986 in the terminal of LEE4. EspF harbors three proline-rich repeats (PRR) in enteropathogenic *E. coli* (EPEC) [10], four PRR in EHEC, and five PRR in *Citrobacter rodentium* [11]. Each PRR comprises two putative overlapping Src homology 3 (SH3) binding domains with the consensus PxxP motif, mediating binding to the well-characterized SH3 domain, and contains a functional neuronal Wiskott-Aldrich syndrome (N-WASP) binding motif [12], [13]. EspF is thought to be able to destroy the barrier of intestinal epithelial cells, and even to target mitochondria through the mitochondrial targeting signal (MTS) area in the N terminal domain, initiating a process of early apoptosis in host cells [8]. According to recent studies, EspF can bind to some host cell proteins by the SH3 and EVH1 domains, which can result in interference with signal transmission [14], [15], [16].

Recently, according to some studies, EspF employs SNX9 to induce the formation of membrane tubules in cytosol during the invasion of intestinal epithelial cells by EPEC [12], [17], [18]. Additionally, the putative N-WASP binding motif of each PRR mediates the direct interaction of EspF with the Cdc42/Rac-interactive binding (CRIB) domain of N-WASP [12]. Subsequently, EspF binds to and activates N-WASP to induce multiple actin polymerization which are regulated by Cdc42 normally and binds cytokeratin 18 to facilitate the collapse of intermediate filament (IF) network of epithelial cells [12], [19], [20]. In EPEC, EspF cooperates with the effectors Map, Tir, EspG, and NleA to mediate rapid tight junction (TJ) disruption. EspF is therefore distinctly multifunctional with many overlapping functions such as targeting mitochondria, disrupting tight junctions, effacing microvilli, inhibiting the water transporter SGLT-1 and inhibiting phagocytosis in EPEC [16], [21], [22], [23], [24], [25]. Thus EspF becomes an emerging feature of effector proteins essential for intimate attachment in EPEC and ruling as a bacterial pathogen’s Swiss army knife [9], [26], [27].

Previous studies in EPEC have demonstrated that EspF acts as a bacterial effector protein. Nevertheless, the molecular pathogenesis of A/E lesions during EHEC O157:H7 infection is still in deciphering. In this study, we show that EHEC EspF translocates into intestinal epithelial cells, targets mitochondria, and induces apoptosis. Compared to the EHEC prototype O157:H7 Guangzhou strain, the EHEC *espF*-deficient mutant (Δ*espF*) we constructed previously had obvious lower bacteria adhesion, lower caspase-9/3 and lactate dehydrogenase (LDH) levels, lower cell attachment and effacement abilities, weaker mitochondria apoptosis, and a higher mouse survival rate.

## Results

### Construction of Δ*espF* Mutants of EHEC O157:H7

An *espF*-deficient mutant in N-terminal domain was constructed by using overlap extension PCR (OL-PCR). The segments A (865 bp) and B (897 bp) of gene *espF* were amplified with two pairs of primers (primers A1, A2 and primers B1, B2, respectively) designed according to the sequence of *espF* and its upstream and downstream ends, respectively. The segment Δ*espF* (1762 bp), harboring the 162 bp knock-out gene, was cloned into a pCVD442 suicide vector resulting in pCVD442-Δ*espF* in *E. coli* SM10λπ. Subsequently, the pCVD442-Δ*espF* was transformed from *E. coli* SM10λπ into wildtype EHEC O157:H7. All EHEC strains were cultured in Luria broth (LB) media supplemented with appropriate antibiotics, nalidixic acid, and ampicillin at 37°C for routine passage. The Nal^R^ and Amp^S^ strains of EHEC O157:H7 (Δ*espF*) were screened by PCR amplification with primers A1 and B2, which resulted in only one band (nearly 2000 bp), whereas two bands were amplified in the EHEC O157:H7 Nal strain (pCVD442-Δ*espF*) by PCR (data not shown). It was also found via DNA sequencing that the Δ*espF* mutant lost 162 bp. The genome location of the deletion was at nt 4659104–4659265, which was within the N-terminal domain of EspF.

### The Δ*espF* Strain had Weaker Adhesive Ability to *Lovo* Cells

**Figure 1:**
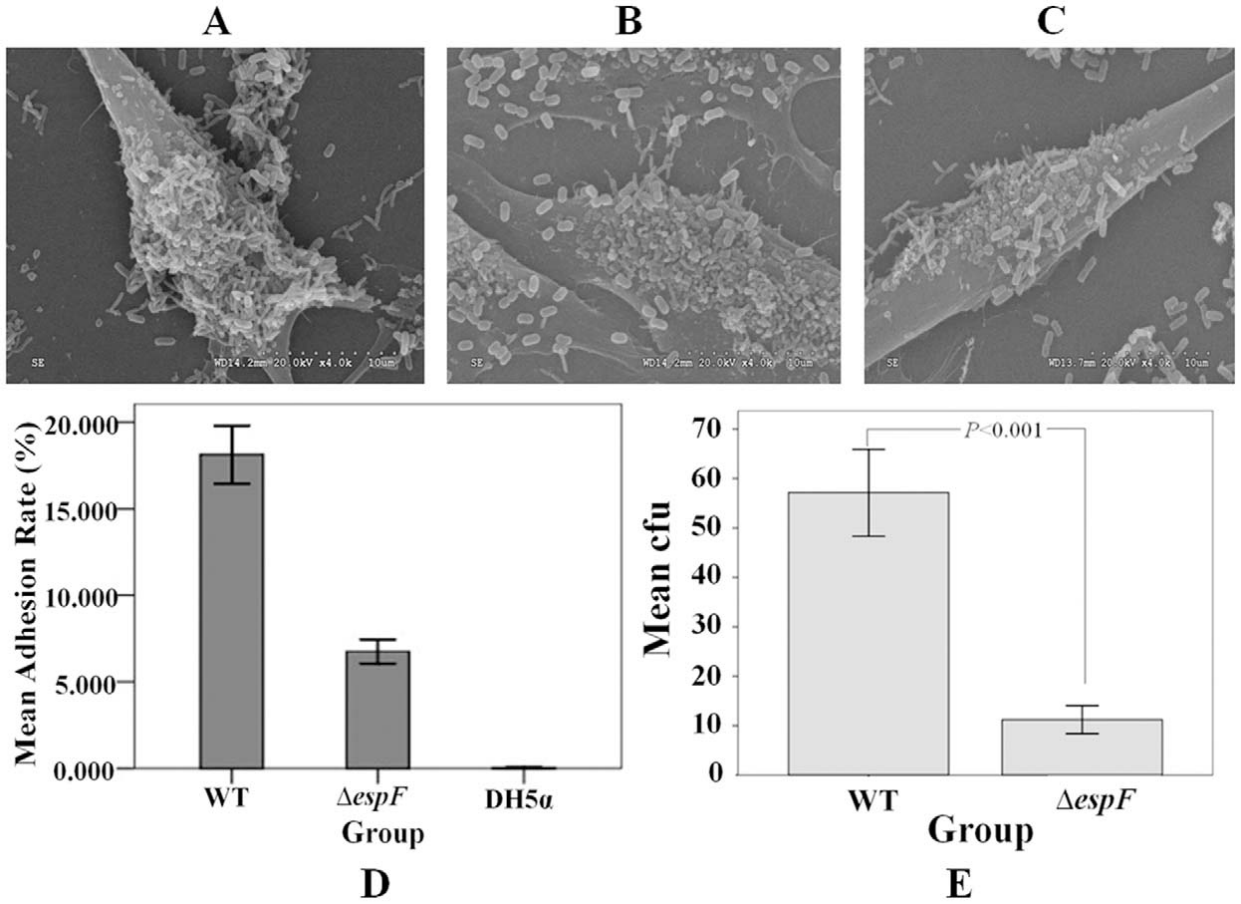
Adhesive ability of EHEC O157:H7 with *Lovo* cells under electron microscopy. (A) *Lovo* cells infected with EHEC O157:H7 wild type (WT) strain. (B) *Lovo* cells infected with EHEC O157:H7 *espF*-deficient mutant (Δ*espF*) strain. (C) *Lovo* cells infected with *E. coli* DH5α. (D) Mean adhesion rate of *Lovo* cells infected with wild type, Δ*espF*, and DH5α groups; results show mean ±SD (n = 3). (E) Mean cfu of *Lovo* cells infected with wild type and Δ*espF*; results show mean ±SD (n = 3); *P*<0.001 for all groups.

*Lovo* cells were infected separately with EHEC O157:H7 wild type (WT), EHEC O157:H7 *espF* mutant (Δ*espF*), or *E. coli* DH5α (DH5α) strains to determinate their bacteria adhesion rates. The membrane of *Lovo* cells was observed via electronic microscopy 3 h post infection with WT, Δ*espF*, and DH5α. Compared with the WT strain’s adhesion rate (18.128±3.159‰), the Δ*espF* strain’s adhesion rate on the cells surface (6.756±1.297‰) was three-fold lower. The average adhesion rate of the DH5α strain was 0.043±0.015‰, resulting in few bacteria on the cell membrane surface. Neither the Δ*espF* nor the DH5α control group infections cause cell membrane damage (Fig. 1). The lower adhesion rate of the Δ*espF* mutant strain indicated that *espF* gene deletion reduced EHEC adhesive capability.

To determine the number of adherent bacteria in mouse colons, mice were infected with WT and Δ*espF* strains twice (2×10^10 ^CFU/mL and 1.5×10^10 ^CFU/mL). Colon sections 5-cm distal from the rectum of each group were collected vertically 7 d post-infection. The colon specimens were then homogenized within 1 mL of ice-cold phosphate-buffered saline (PBS) after being removed fecal pellets. LB agar plates were used to calculate the amount of bacteria. Figures 1D and 1E revealed that the Δ*espF* group had significantly fewer adherent bacteria (11.20±4.022) than the WT group (57.10±12.342) (*t = *11.182, *P*<0.001).

### The EHEC Effector Protein EspF Induces *Lovo* Cell Apoptosis Post EHEC Infection

**Figure 2:**
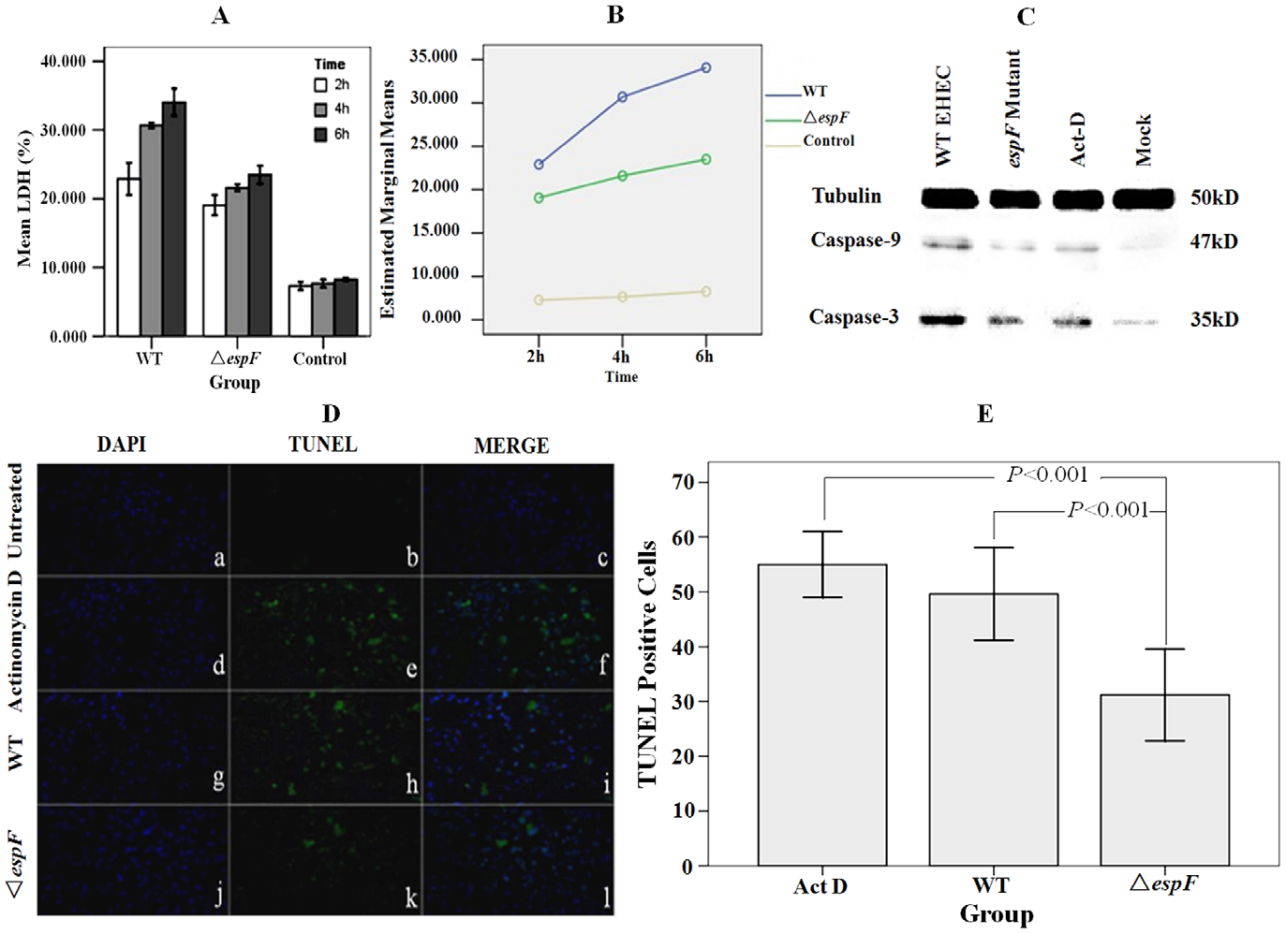
The EHEC effector protein EspF induces *Lovo* cell apoptosis post EHEC infection. (A) LDH release rate comparison of *Lovo* cells infected with wild type, Δ*espF*, and DH5α groups at 2, 4, and 6 h; results show mean ±SD (n = 3). (B) Estimated marginal mean of LDH release rates of wild type, Δ*espF*, and DH5α groups. (C) Detection of caspase-9/3 in infected *Lovo* cells. Cells were untreated (mock), treated with EHEC WT, Δ*espF* strains and the apoptosis inducer actinomycin D (Act-D) respectively for 3h. (D) TUNEL test fluorescent images of *Lovo* cells infected with EHEC. (b, e, h, k): cells untreated, treated with Act-D, infected with EHEC WT, Δ*espF* respectively, and immunostained with TUNEL. (a, d, g, j): cells counterstained with DAPI and observed based on fluorescence microscopy. (c, f, i, l): merged images of TUNEL and DAPI treated cells. (E) Quantification of TUNEL-positive cells. Dead cells were stained by TUNEL assay and TUNEL-positive cells were counted based on fluorescence microscopy; results showed mean±SD (n = 3); *P*<0.001 for all groups.

Lactate dehydrogenase (LDH) variation was determined in *Lovo* cells at 2, 4, and 6 h post-infection by WT, Δ*espF*, and DH5α (control) strains (Fig. 2A). The LDH release rate in the Δ*espF* group averaged 21.385%, which was lower than WT group (29.214%). The DH5α control group had the lowest LDH release rate. LDH release increased coupled with the increasing infection time. The marginal means of LDH release rate were significantly different for both time and strain groups (Fig. 2B) (*F* = 34.619, *P*<0.01, and *F* = 113.305, *P*<0.01, respectively).

Cysteine-requiring aspartate protease (caspase) is a protease family that plays an important role in the process of cell apoptosis. Caspase-9 is an upstream protease in the process of the cell apoptosis signal transduction, which is activated and regulated by phosphorylation [28]. Caspase-9 activates the key enzyme caspase-3 during cell apoptosis in order to promote follow-up cell apoptosis signals. Caspase-3 is one of the key executioner molecules in cell apoptosis and cleaves several substrates that indicate apoptotic death [29]. Using antibodies that recognize caspase-9/3, we performed western blotting to detect procaspase-9/3 expression of *Lovo* cells’ post infection with WT and Δ*espF* strains. Three hours post infection, equal amount of cells were lysed and SDS-PAGE was performed to screen the proteins. Tubulin was used as an internal protein control. Cells treated with actinomycin D (Act-D) were as a positive control. The results showed that little caspase-9/3 was detected in non-infected cells (Fig. 2C). A trace amount of caspase-9/3 was found in the Δ*espF* infected group, whereas the WT infected group had a higher caspase-9/3 expression level. The results showed that EHEC EspF influenced in the expression of caspase-9/3, indicating an association with the cell apoptosis pathway.

The terminal deoxynucleotidyl transferase dUTP nick end labeling (TUNEL) assay involves fluorescein labeled dUTP staining of fragmented DNA 3′-OH ends of apoptotic cells identified by terminal deoxyribonucleotidyl transferase (TdT). Because of the sparseness of DNA fragments and lack of 3′-OH in healthy cells, there is no fluorescent signal emitted when labeling by fluorescein dUTP. Since EspF can initiate the apoptosis pathway, we used TUNEL staining and DAPI counterstaining to visualize *Lovo* cell apoptosis post infection with EHEC WT, Δ*espF*, and Act-D, separately. Observations were made via fluorescence microscopy (Fig. 2D). In the DAPI group, we noted that the non-infected cells group emitted blue fluorescence, but no green fluorescence (Fig. 2D: a). In the TUNEL group, cells infected by EHEC WT and Act-D emitted numerous green fluorescent signals (Fig. 2D: e and h); however, cells infected by Δ*espF* emitted few green fluorescent signals (Fig. 2D: k). In the merged groups (Fig. 2D: c, f, i, l), cells infected by EHEC WT and Act-D distinctly emitted green fluorescence, indicating the presence of marked cell apoptosis. On the contrary, cells infected by Δ*espF* revealed less green fluorescence, indicating less cell apoptosis. The TUNEL assay results allowed determination of the amount of TUNEL-positive cells: 49.60±6.804 cells in the EHEC WT group, similar to the 55.00±4.848 cells in the Act-D group. The Δ*espF* group had the fewest cells (31.20±6.760) (Fig. 2E). A least significant difference (LSD) method analysis revealed a significant difference (*F* = 20.220, *P = *0.000<0.001) among these groups. In summary, the WT and Act-D groups had more apoptosis, as indicated by larger amounts of green fluorescence, than that in the Δ*espF* group. The mild green fluorescent signals in the Δ*espF* group indicated that Δ*espF* strain has a deficient capability to induce host cell apoptosis.

### EspF Targets Host Cellular Mitochondria via TTSS

The DAPI is known to form blue fluorescent complexes with natural double-stranded DNA, showing fluorescence specificity for A-T regions of DNA. When DAPI binds to DNA, the blue fluorescence is enhanced greatly. Thus, cell nucleus staining with DAPI serves to provide a cell localization function. Mitochondria heat shock protein 70 (mtHsp70) is located in the matrix of mitochondria, and we used it to show indirectly the location of mitochondria. For localization study, anti-EspF, marked with fluorescein isothiocyanate (FITC) and emitting a green fluorescent signal, and anti-mtHsp70, marked with Rhodamine and emitting a red fluorescent signal were used.

**Figure 3:**
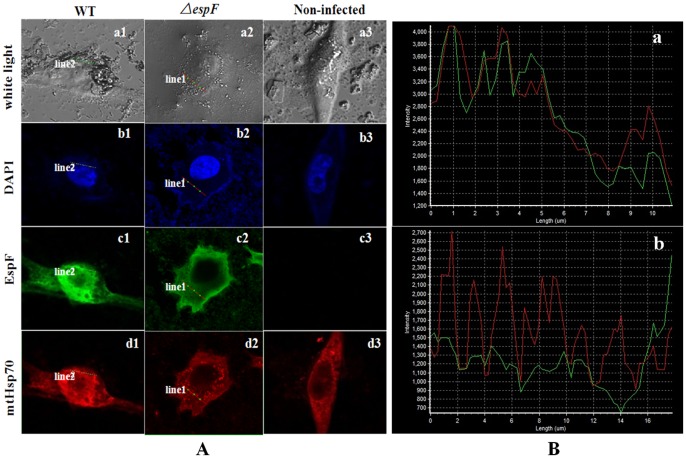
The EHEC effector protein EspF targets the mitochondria post infection. (A) Confocal laser scanning microscopy of *Lovo* cells infected with EHEC O157:H7. Confocal through the mitochondria of cells: EHEC O157:H7 WT group, Δ*espF* group and control group. (a1–a3): the observation under white light based on confocal microscopy. (b1–b3): blue fluorescence signal from the DAPI stained nucleolar region. (c1–c3): green fluorescence signal from the FITC stained EspF protein. (d1–d3): red fluorescence signal from the Rhodamine stained mitochondria heat shock protein 70 (mtHsp70). (B) Red curve enclosed the fluorescent signal of rhodamine stained mtHsp70. Green curve revealed the fluorescent signal of FITC stained EspF protein. (a): curve of WT group. (b): curve of Δ*espF* group.

The MOCK cells and cells infected with WT and Δ*espF* strains were stained and visualized using confocal laser scanning microscopy (Fig. 3A). For the EHEC O157:H7 WT group, both the green fluorescent signal from EspF protein and the red fluorescent signal from mtHsp70 were strong at corresponding locations (Fig. 3A), indicating a positive correlation between the locations and the two proteins. However, for the Δ*espF* mutant, in which the EspF protein green fluorescent signal is weak, the mtHsp70 red fluorescent signal was either strong or weak in corresponding locations; that is, there was no location correlation between the two proteins. Moreover, there was no green fluorescence in the non-infected cells group (Fig. 3A: c3 and d3). This shows that the ability of EspF to target mitochondria was reduced in the Δ*espF* mutant.

In order to further study the relationship of EspF and mtHsp70, we scanned EspF’s green fluorescent signal and mtHsp70’s red fluorescent signal along a straight line scan (Fig. 3B). Along the linear scan of the WT strain, the green fluorescent signal and red fluorescent signal curves revealed a notable synclastic tendency (Fig. 3B: a). This positive correlation showed obvious co-orientation and a strong conjunction between EspF protein and mtHsp70. However, in the Δ*espF* strain, the signal curves manifested a reversed tendency (Fig. 3B: b). The weak green fluorescent signal and strong red fluorescent signal revealed a negative correlation and a reduced conjunction between the two proteins (Fig. 3B: b). In contrast to the strong conjunction between EspF protein and mtHsp70 in EHEC WT, the weak conjunction in Δ*espF* illustrated that EspF targets mitochondria. This is consistent with observations of laser confocal scanning microscopy (Fig. 3A).

### The Δ*espF* Mutant has a Weaker Ability to Induce Mitochondrial Apoptosis

**Figure 4:**
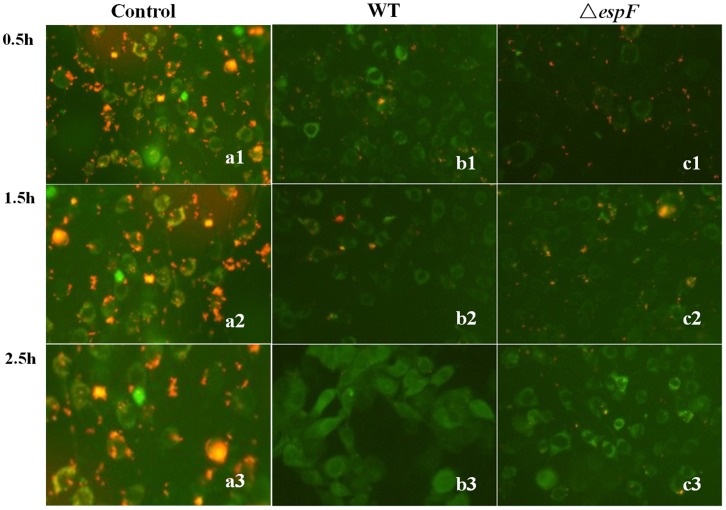
Mitochondrial membrane potential detection indicates cell apoptosis. Green fluorescence reveals the JC-1 stained mitochondrial plasma with a low JC-1 concentration (monomer) that mitochondrial membrane potential depolarized. Red fluorescence encloses the JC-1 stained mitochondrial plasma with a high JC-1 concentration (polymer). (a1–c1): mitochondria of *Lovo* cells that were infected with WT, Δ*espF* and control group for 0.5 h. (a2–c2): mitochondria of *Lovo* cells that were infected with WT, Δ*espF* and control group for 1.5 h. (a3–c3): mitochondria of *Lovo* cells that were infected with WT, Δ*espF* and control group for 2.5 h.

As the results shown above, EspF targeted host mitochondria, but they do not show the mitochondrial apoptosis path. The cationic, lipid fluorescent dye 5,5′,6,6′-tetrachloro-1,1′,3,3′-tetraethylbenzimidazolcarbocyanine iodide (JC-1) was used to detect the potential across a mitochondrial membrane. There are two types of JC-1 in stained mitochondrial plasma, one is a monomer, which emits green fluorescence in a low JC-1 concentration area, and the other is a polymer, which emits red fluorescence in a high JC-1 concentration area. Depending on the potential (Δψ) of the mitochondrial membrane, JC-1 transfers into the mitochondria, which results in a high JC-1 concentration with a red fluorescence. When cells undergo apoptosis, due to mitochondrial membrane potential depolarization, JC-1 is released from mitochondria, which leads to a low JC-1 concentration and a green fluorescence. Changes in mitochondria membrane potential and fluorescent signals in *Lovo* cells were observed at 0.5, 1.5, and 2.5 h post infection with EHEC WT and Δ*espF* strains (Fig. 4). In the WT group, at 1.5 h post infection, red fluorescent signals reduced and green fluorescent signals increased. At 2.5 h post infection, red fluorescent signals had almost disappeared and green fluorescent signals were greatly enhanced. In the Δ*espF*-infected group, at 0.5 h, there were stronger red fluorescent signals and weaker green fluorescent signals compared with the WT group. At 1.5 h, there was little change in either of the fluorescent signals, while at 2.5 h, there were fewer red fluorescent signals remaining but stronger green fluorescent signals. Overall, the results indicated that cell apoptosis is more obvious in WT-infected group than in Δ*espF*-infected group at 0.5 h, while at 2.5 h there is more deterioration of apoptotic cells in a WT-infected group than that in a Δ*espF*-infected group. Above all, the Δ*espF* mutant strain has a weaker ability to induce mitochondrial apoptosis.

### The Δ*espF* Strain has the Weaker Virulence in BALB/c Mice

**Figure 5:**
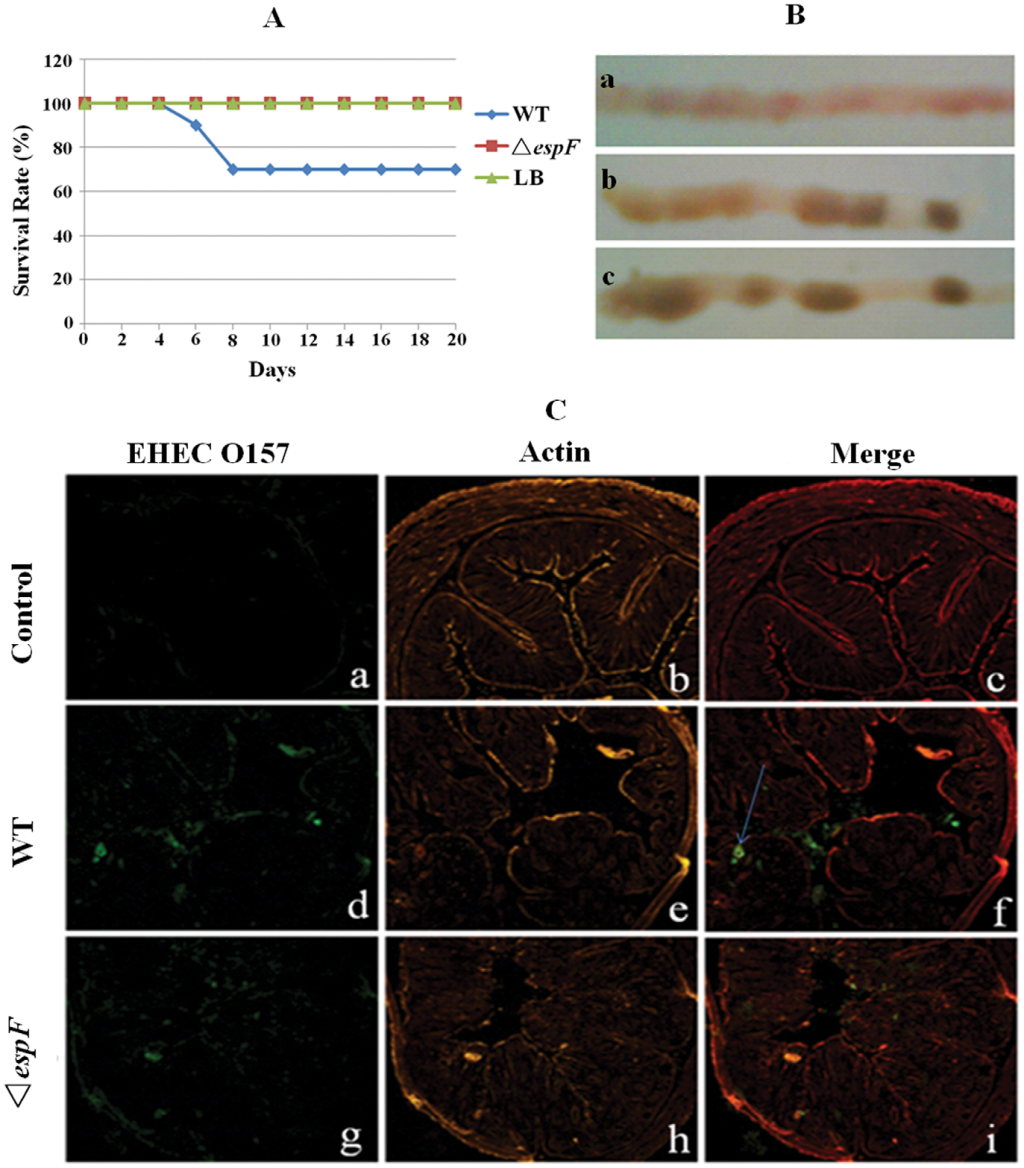
The Δ*espF* mutant strain reduces the pathological injury of mouse. (A) Survival curves of BALB/c mice infected with EHEC strains. (B) Typical features of colons after infection. (a)**:** typical intestinal colitis symptoms in the WT group. (b): healthy colons with slightly swollen areas in the Δ*espF* group. (c): healthy colons with solidified feces in control group (mice were fed by LB). (C) Immunofluorescent images of mouse colons infected with wild type group, Δ*espF* group and control group. (a, d, g): green fluorescence from immunostainning with anti-EHEC O157 antibody. (b, e, h): orange fluorescence from the actin counterstainning with Rhodamine-phalloidin. (c, f, i): merged images of immunostainning with anti-EHEC O157 antibody and Rhodamine-phalloidin stained colons.

Post-infection with EHEC O157:H7, mice exhibited a series of symptoms such as anorexia, lethargy, hind limb atony, quadriplegia, and even death. Mice were divided into three groups and were treated with WT EHEC, Δ*espF* and LB (control), respectively. In order to improve their infection susceptibility, mice were administrated mitomycin C (MMC, 2.5 mg/kg) by intraperitoneal injection and nalidixic acid (Nal, 50 µg/L), by water-feeding, which restrained the intestinal flora level [30]; Simultaneously, they were infected with EHEC WT or Δ*espF* strains. In the EHEC WT group, BALB/c mice were inoculated orally. At day 3 post-infection, some symptoms appeared such as slow-movements, listlessness, anorexia, and hair fleeciness. Some began twitching and exhibiting hind limb atony in the following days. At day 7 post-infection, 3 mice died (mortality rate 30%, Fig. 5A) and the symptoms of the mice were most severe. Subsequently, the symptoms of the other mice left gradually recovered day after day. In the Δ*espF* mutant group, mice were also inoculated orally and, at day 4 post-infection, some mice exhibited rage, listlessness, and slow-movements. From days 6–7 post-infection, all mice began to recover gradually. In the WT group, mice had diarrhea at days 2–3 post-infection; however, mice in the Δ*espF* group had no significant diarrheal symptoms. Throughout the post-infection period, the control group mice were healthy. The results demonstrated that the Δ*espF* mutant strain has a weaker virulence compared to EHEC O157:H7 WT.

BALB/c mice infected with EHEC WT, Δ*espF* strains and, LB were executed at day 7 post-infection. Sections of colon 5-cm distal from the rectum were collected. Typical pathological features of the colon sections were observed. The sections were weighed and the numbers of adhesive bacteria were counted before the sections were frozen. Colon sections from the WT group exhibited typical, edematous and inflammatory, intestinal colitis with a few solidified feces. However, sections from the Δ*espF* group appeared healthier with solidified feces, but some portions appeared slightly swollen. In the LB control group, the colon sections were healthy with solidified feces (Fig. 5B: a, b and c).

In addition, other parts of the colon sections of mice infected with WT and Δ*espF* strains were fixed with 4% paraformaldehyde and frozen. Colonic mucosal adhesive bacteria within the frozen sections were immunostained with anti-EHEC O157 antibody. As well, the actin of the colonic epithelial cells were counterstained with Rhodamine-phalloidin and observed through fluorescent microscopy. Stained sections from the LB control group revealed no green fluorescence whereas the Δ*espF* group sections revealed little fluorescence; however, strong fluorescent signals appeared over the epithelial surfaces, even in intestinal crypts, in the WT group (Fig. 5C: a, d, g). Observed by counterstaining with Rhodamine-phalloidin, the colonic epithelial cell cytoskeletal structure, microvilli, and bowel pitch were complete and healthy in the LB group (Fig. 5C: b). Sections from the Δ*espF* group showed mild lesions (Fig. 5C: h); however, sections from the WT group showed various lesion symptoms such as enteric cavity changes, intestinal crypt absences, and effaced microvilli (Fig. 5C: e).

The above experimental results showed that the Δ*espF* mutant has a weaker virulence and induces milder A/E lesions than WT EHEC. The mild microvilli effacement and reduced cells apoptosis indicates an important role of EspF in colonization of the intestinal epithelium and cell apoptosis initiation.

## Discussion

As has been reported, once adhered to intestinal epithelial cells via intimin, EHEC and EPEC inject secretion effectors, such as Tir, Map, EspG, EspF, and EspH, into host cells through the TTSS [6]. This results in disrupting the epithelial barrier, effacing microvilli, remodelling host membranes, and leading to mitochondrial dysfunction [8]. In this study, we constructed an EHEC O157:H7 *espF*-deficient (Δ*espF*) strain with a 162 bp (4659104–4659265) deletion in the N-terminal domain, by using OL-PCR. Subsequently, we compared *Lovo* cell apoptosis, colonic epithelia A/E lesion processes and also mice infections between WT and mutant strains. The results indicated the influences of *espF* N-terminal deficiency on cell attachment and effacement abilities, caspase-9/3 and lactate dehydrogenase levels, mitochondria apoptosis, and mouse survival rate.

It has been demonstrated that EspF is multifunctional in many cellular processes. EspF bonds with mitochondria, N-WASP, SNX9, Abcf2, CK18, SGLT-1, and NHE3 to disrupt the epithelial TJ, efface microvilli, modulate the cytoskeleton, induce actin polymerization, recruit the pedestal, and finally lead to apoptosis and A/E lesions [26]. During infection, EspF rapidly accumulates in mitochondria with such accumulation dependent on a functional mitochondrial membrane potential (MMP) [15], [16], [21].

Previous studies showed that the N-terminal domain of EspF in EPEC can target mitochondria in infected host cells [15], [16]. Corresponding to the structure of EspF, the mitochondria binding region (1–101aa before the N-terminal) contains an MTS consisting of an α-helix, a hydrophobic part, and a hydrophilic part, that recognizes the outer membrane’s Tom20, which orientates EspF to the mitochondria. After EPEC adhesion to host cells, EspF first targets mitochondria through the TTSS, then gathers in the cell nucleus [14], [15]. In our study, strong EspF green and mtHsp70 red fluorescent signals indicated co-orientation of those two proteins (Fig. 3). However, our results displayed a strong green fluorescent signal for the EspF protein and a weak mtHsp70 red fluorescent signal in the Δ*espF* group, indicating no orientation correlation and a reduced mitochondrial binding affinity in the Δ*espF* strain. The non-infected control group did not display any green fluorescence signal. We scanned a straight line of the EspF green and the mtHsp70 red fluorescent signals of each group and a consistent fluorescent signal strength curve was detected in the WT group. The presence of an uncorrelated fluorescent signal strength curve in the Δ*espF* group indicated that EHEC EspF targets mitochondria. Thus, the deletion of N-terminal domain of *espF* gene reduces the mitochondrial binding affinity of EHEC.

The reduction of mitochondrial transmembrane potential initiates cell apoptosis. In *Lovo* cells infected for 0.5 h with WT a reduced red fluorescent signal and an intensive green fluorescent signal revealed an intensive fluorescent change. In contrast, *Lovo* cells infected for 0.5 h with Δ*espF* displayed a strong red fluorescent signal and a weaker green fluorescent signal. At 1.5 h post-infection, the red fluorescence reduced gradually and green fluorescence strengthened gradually in the WT group, whereas both fluorescent signals changed mildly in the Δ*espF* group. At 2.5 h post-infection, red fluorescence in WT almost disappeared while green fluorescence enhanced intensively. And the retention of the red fluorescent signals in Δ*espF* at 2.5 h indicated a milder apoptosis than in WT EHEC, which showed a reduced virulence of the Δ*espF* strain. All of these results showed that the N-terminal deficiency of EspF induces weaker mitochondria apoptosis.

Caspase is a protease family that has an important role in cell apoptosis and it has activity sites, which specifically cleave the procaspase polypeptide of aspartic acid residues. Caspase cleaves some proteins selectively, which induces cell apoptosis [31]. Foreign protein signals cleave and activate the initiator caspases and the executioner caspases subsequently. The executioner proteolysed caspases lead to the morphological and biochemical characteristics of apoptosis. Caspase-9 is the initiator caspase in the “intrinsic” or mitochondrial caspase pathway and is activated in response to cytochrome c release from the mitochondria of pre-apoptotic cells [29]. Activated caspase-9 cleaves and activates the apoptosis executioner caspase-3, which promotes the subsequent apoptosis signal. Serving as an important index of apoptosis, caspase-3 is one of the key executioners of cell apoptosis that cleaves many key proteins. Our results from *Lovo* cells infected with EHEC WT and Δ*espF* showed that both WT and Δ*espF* leaded to caspase-9/3 expression, with WT expressing more caspase-9/3 than expressed by Δ*espF.* This suggests that Δ*espF* has a lower virulence and is able to reduce the level of apoptotic proteins. In each group, caspase-3 was expressed more than caspase-9, indicated a late stage of apoptosis. Compared with our negative control, caspase-9/3 release in the Δ*espF* strain was slightly more than that in a natural release, indicating that the Δ*espF* mutant retained some virulence.

Mice are ideal animal models for use in EHEC O157:H7 pathogenesis research [32], [33]. Vallance *et al.* inoculated mice orally with wild type *Citrobacter rodentium* (CR) to research the pathogenesis of A/E bacteria and found that CR infections vary among different mouse strains; for example, C3H/HeJ, C57BL/6, and BALB/c mice were more susceptible to bacteria invasion and colonization than other strains [34]. The study of Fujii *et al.* verified that some mice were able to live following administration of 10^10^ EHEC O157:H7 bacteria [30]. However, given an intraperitoneal injection of MMC and fed streptomycin water during bacterial inoculation can increase the susceptibility of mice. MMC induces a decrease in white blood cells and platelets, which is similar to the results of cyclophosphamide treatment. In addition, streptomycin restrains the normal intestinal flora, which can result in EHEC O157:H7 being the dominant member of the flora [35], [36], thus, enhancing the EHEC O157:H7 susceptibility of mice. So, in this study, BALB/c mice were selected as the model and injected with MMC intraperitoneally and fed with nalidixic acid water during the period of infection with EHEC O157:H7. The results showed that the Δ*espF* strain had weakened EHEC virulence, bacterial infection, and pathogenicity toward mice. The immunofluorescence observations of colon epithelial cell damage showed typical colitis symptoms (colon edema, sparse feces, and little obvious granular dung) in the EHEC WT group. However, HC symptoms were not observed, indicating that A/E lesions were present, but not very serious. This lack of hemorrhagic symptoms may correlate to the susceptibility of the mice and the virulence of the bacteria. In the Δ*espF* and the LB groups, colons remained healthy throughout the infections with no edema and granular feces, demonstrating that an *espF* gene deficiency weakens EHEC lesions and adhesion.

In conclusion, as shown in the results, an *espF*-deficient mutant of EHEC O157:H7 is characterized by a low bacterial virulence, reduced adhesive capability and mitochondrial apoptosis, and milder A/E lesions. In contrast to its actions in EPEC, EspF of EHEC targets mitochondria with no evidence of its aggregation in cell nuclei. Furthermore, we showed that the EspF N-terminal domain of EHEC O157:H7 induces cell apoptosis. Our results revealed that the EspF protein in EHEC O157:H7 induces host cell apoptosis and leads to A/E lesions in intestinal epithelial cells. These findings have important scientific significance to further the elucidation of the molecular pathogenesis of EHEC O157:H7.

## Materials and Methods

### Ethics Statement

This study was carried out in strict accordance with the recommendations in the Guide for the Care and Use of Laboratory Animals of the National Institutes of Health. The protocol was approved by the Committee on the Ethics of Animal Experiments of the Southern Medical University (Permit Number: 2009-052). All surgery was performed under sodium pentobarbital anesthesia, and all efforts were made to minimize suffering.

### Bacteria, Plasmids, and Mammalian Cell Culture

The EHEC O157:H7 strain GZ-246 was isolated from the Guangzhou Centers for Disease Control Prevention and grown in LB broth media at 37°C for routine passage. Before infection, *Lovo* cells were cultured in DMEM (Hyclone) containing 10% FBS and 1% penicillin/streptomycin in 5% CO_2_ for routine passages. Agar (MDBio) was added to obtain 1.5% (agar plates, m/V) as indicated.

### Antigen Design and Synthesis

The Hopp-Woods and Kyte-Doolittle schemes within Protean software (DNAStar) were performed to forecast the hydrophilic amino acids [37], [38]. The Karplus-Schultz and Emini methods were performed to forecast flexibility and surface possibility [39]. The Jameson-Wolf and Wu’s methods were performed to forecast the potential B cell epitope [40]. The superior region of the B cell epitope was selected and conjugated to keyhole limpet hemocyanin (KLH) by using Cys in its C-terminal. The antigen was synthesized by Shanghai Jill Biochemical.

### Generation of *espF*-deficient EHEC O157:H7 Strain

To obtain the Δ*espF* mutant with a 162-bp fragment knock-out, EHEC O157:H7 genomic DNA was isolated using a bacterial genomic DNA Extraction Kit (TIANGEN) and was as a PCR template. The *espF-deficient* gene (Genbank accession number NC_002655, GI:12518435) was amplified twice by using overlap PCR with a high-fidelity *Taq* DNA polymerase (TaKaRa) and primers A1 (5′-GGCGGCTCTAGATTATCGCTGACTCAT-3′), A2 (5′-CCGCCCTAGTGTAGAAGCA-3′) as well as primers B1 (5′-TGCTTCTACACTAGGGCGGACTTCATTTACTC-3′), B2 (5′-GCCGAGCTCTGA AGCCATCTAAGT-3′). The second-round PCR was ran with primers A1 (5′-GGCGGCTCTAGATTA TCGCTGACTCAT-3′) (containing an *XbaI* site) and B2 (5′-GCCGAGCTCTGAAGCCATCTAA GT-3′) (containing a *SacI* site) by using the previous PCR product as templates. In total, there were 30 cycles in the PCR process. Both PCR products were purified by using Gel Extraction Kits (TaKaRa). Purified Δ*espF* was cloned into the pMD19T vector (TaKaRa) and transformed into *E. coli* DH5α competent cells. Subsequently, it was subcloned into pCVD442 (TaKaRa) and transformed into *E. coli* SM1010λπ (A gift from Dr. Shenghe Huang). The resulting clones were screened by Amp antibiotics and by PCR with primers A1 and B2. The *E. coli* SM1010λπ strains containing pCVD442-Δ*espF* were cultured in LB broth (100 µg/mL Amp) at 37°C overnight.

EHEC O157:H7 was grown in LB agar plates at 37°C and a spontaneous nalidixic acid resistant mutant named EHEC O157:H7(Nal^R^) was selected for culture in LB broth (50 µg/mL Nal) at 37°C overnight. A mixture of EHEC O157:H7(Nal^R^) and *E. coli* SM1010λπ containing pCVD442-Δ*espF* at 1∶2 (v/v) was centrifuged at 6000 r/min for 5 min, followed by placement on Nal and Amp LB agar plates at 37°C overnight to screen for Nal- and Amp-resistant strains. Then, the bacteria EHEC O157:H7 (pCVD442-△*espF*) were diluted and cultured on 5–10% sucrose selection plate to obtain the Amp-sensitive strain. The resulting mutant strain, named EHEC O157:H7 Δ*espF*, was verified by using PCR screening followed by DNA sequencing (Invitrogen).

### EspF Polyclonal Antibody Production

Two New Zealand white rabbits (each weighing 1.5–2.5 kg) were selected for the collection of venous blood (2 mL) from the auricular margin before infection. Venous serum was obtained and kept in −20°C as a control. Antigen protein was dissolved by normal saline to immunize the rabbits at days 1, 28, 42, and 66 at 1 mg per rabbit. Initially, antigen protein was emulsified in an equal volume of Freund’s complete adjuvant. Each rabbit was subcutaneously injected with 2 mL of antigen at multiple points along the back and shoulder. As an immunization boost, antigen protein emulsified with Freund’s incomplete adjuvant was intramuscularly injected as 2 mL of antigen at the shoulders and legs of each rabbit. Post-boost, venous blood (1 mL) from the auricular margin was collected for antibody valence testing by ELISA. Ten days post-boost, cardiac whole blood was collected and antibody valence in the serum was determined by ELISA. Antiserum specificity was tested by western blot. Subsequently, polyclonal antiserum was purified by caprylic acid-ammonium sulfate precipitation and its purity was detected by SDS-PAGE.

### Western Blot Detection

*Lovo* cells were infected with EHEC O157 WT and Δ*espF* strains. Three hours post-infection, cells were washed with phosphate-buffered saline (PBS, 0.01 mol/L, pH 7.4) three times and then lysed. Protein was extracted and screened by 15% SDS-PAGE electrophoresis (40 µL/hole). The electrophoresis band was then transferred to a PVDF membrane. Pre-immunization and post-immunization sera as primary antibodies were diluted at a rate of 1∶1000 and incubated overnight at 4°C. The primary antibodies were then washed three times (5 min per wash). Goat anti-rabbit IgG/HRP diluted at a rate or 1∶5000 was used as a second antibody. The results were observed by a gel imaging apparatus.

### Bacteria Adhesion Test

*Lovo* cells were incubated on slides, infected with EHEC for 2 h dyed with Giemsa stain, dried, and then observed by microscopy. After 12 h of culture, WT, Δ*espF*, and DH5α (negative control) strains were suspended in DMEM (10% FBS and no antibiotics). *Lovo* cells were seeded in 12-well tissue culture plates. *Lovo* monolayer cells (about 2×10^5^ cells per well) were infected with EHEC (2×10^7^ bacteria per well) and cultured at 37°C in 5% CO_2_ for 1.5–2 h. The cells were washed three times with PBS then lysed using 150 µL 0.5% Triton X-100 for 8 min. Subsequently, 100 µL PBS was added and mixed. Finally, colonies on two plates were counted after gradient dilution and the adhesion rate was calculated (adhesion rate = adhered bacteria per well/initial bacteria per well×1000%). Results were analyzed by using the One Way ANOVA of SPSS13.0 software.

Mice were sacrificed at day 7 post-infection with EHEC WT or Δ*espF* or feeding LB as a control. The sections of colon 5 cm distal from the rectum were collected from mouse rectums and weighed. Specimens from WT- and Δ*espF*-infected mice were ground with 1 mL ice-cold PBS. The homogenates were then serially diluted with ice-cold PBS and seeded on LB agar plates that contained 50 µg/mL nalidixic acid. The number of colony-forming units (CFU) per mouse was then calculated.

### Lactate Dehydrogenase (LDH) Release Analysis

*Lovo* cell monolayers (1.5–2×10^5^ cells per well) infected with EHEC O157:H7 WT and Δ*espF* strains (2×10^7^ bacteria per well) were incubated at 37°C in 5%CO_2_ for 2, 4, and 6 h. Plates were then transferred to a microplate reader (BioTek), excited at 490 nm, and the emission signals read at 490 nm and at 650 nm as a reference. All tests were performed in triplicate and the amount of LDH released was calculated as described previously [41].

### TUNEL Assay

*Lovo* cells were seeded in dishes, then infected and cultured as described above. After 3 h of infection, cells were fixed with PBS containing 4% paraformaldehyde, sealed with methanol containing 3% H_2_O_2_, and permeated with 0.1% sodium citrate solution containing 0.1% Triton X-100. The pretreated cells were stained by using a One Step TUNEL Apoptosis Assay Kit (KeyGEN) and held at 37°C under moist dark conditions for 1 h. Nuclei were counterstained by 4, 6-Diamidino-2-phenylindole (DAPI; Sigma) for 5 min under the same conditions. Results were observed by a fluorescent microscopy (Axioplan 2, Zeiss). TUNEL-positive cells of at least five fields in view were counted at magnification (20×10).

### Caspase-9/3 Detection

*Lovo* cells were infected with EHEC O157:H7 WT, Δ*espF*, and actinomycin D (Act-D) and incubated at 37°C in 5% CO_2_ for 3 h. Subsequently, the cells were washed gently with ice-cold PBS, lysed by RIPA lysis buffer (50 mM Tris pH 7.4, 150 mM NaCl, 0.5 M EDTA, 1% Triton X-100, 1% sodium deoxycholate, 0.1% SDS, sodium fluoride, and 1 mM phenylmethylsulfonyl fluoride [PMSF]) on ice for 10 min. The supernatant fluid was extracted by centrifugation (12,000 r/min for 5 min) at 4°C and the obtained proteins were screened by SDS–PAGE. Immunoblotting with mouse monoclonal anti-caspase-9 antibody (BioLegend/Biocompare) and mouse monoclonal anti-caspase-3 antibody (Abcam) was performed as described above. In addition, α-Tubulin, as an internal reference to determine the consistent loading quantities of each group, was detected by using anti-tubulin antibody (Biovision). The results were determined by using a multifunction gel imaging system (FluorChem Q).

### EspF Target Assay

*Lovo* cells were cultured in confocal Petri dishes for 12 h and infected with EHEC WT and Δ*espF* at a 1∶100 rate at 37°C for 2 h. Cells were washed with PBS three times, then fixed by polyphosphate formaldehyde (4%) for 30 min and permeated by 0.05% Triton for 20 min. Subsequently, specimens were washed twice with PBS and blocked by PBS containing 1% BSA for 1 h. Specimens were incubated with anti-mtHsp70 antibody and anti-EHEC O157 polyclonal antibody at a rate of 1∶50 at 4°C for 18 h, then washed again with PBS three times, followed by incubation with anti-rabbit IgG-Fitc and anti-rat IgG-Rhodamine at a rate of 1∶50 in the dark for 45 min. Finally, the specimens were again washed with PBS three times, incubated with DAPI for 3–5 min at room temperature, and washed with PBS twice. Results were observed through a laser confocal microscope.

### Detection of Mitochondrial Membrane Potential Variation

*Lovo* cells were incubated in 24-well cell culture plates at 37°C. Twelve hours post-culture, cells were infected with EHEC WT or Δ*espF* for 0.5, 1.5, and 2.5 h, respectively. Subsequently, cells were washed three times with PBS. A JC-1 Kit was used to detect the mitochondrial cross membrane potential. Results were observed through a fluorescent microscope.

### Survival Assay for Mice Infected with EHEC O157:H7

Thirty BALB/c mice (4–5 weeks old, 13.27±1.27 g weight) were randomly divided into three groups (EHEC WT, Δ*espF*, and control groups), with 10 in each group. They were provided water containing nalidixic acid (50 µg/mL) before infection. WT and Δ*espF* strains (approximately 10^10^ CFU/mL) were cultured overnight in 5 mL LB broth containing 50 µg/mL of nalidixic acid at 37°C. A secondary infection began at an interval of 12 h. Ten mice were administered with 500 µL of bacterial suspension (1×10^10 ^CFU/mouse). Simultaneously, they were intraperitoneally injected with mitomycin C (MMC; 2.5 mg/kg). Control group mice were treated with MMC and inoculated equivalently with LB broth under the same conditions. Survival rate was assessed daily for 20 d. The animals were monitored at least four times per day. The food, water, temperature and humidity were monitored for these animals. We changed the bedding frequently during the survival study and also supplied enough food and water, keeping the temperature and humidity constant and suitable. Mice were sacrificed humanely by diethyl ether anesthesia and cervical dislocation when their symptoms became too severe.

### A/E Lesion in Mouse Colon Epithelial Cells

Five mice of each infection group, and inoculated in the same manner, were sacrificed after infection with EHEC WT and Δ*espF* for 7 d. The sections of colon 5 cm distal from the rectum were collected and observed. Colons were weighed after feces elimination via PBS washing. The homogenates were serially diluted with ice-cold PBS, seeded on LB agar plates containing nalidixic acid (50 µg/mL), and cultured overnight at 37°C. The CFU per mouse was then determined. For histopathological analysis, colons infected with EHEC at day 7 post-infection were fixed with PBS containing 4% paraformaldehyde at 4°C for 48 h. The colons were then immersed in a 30% sucrose solution, dehydrated in ampoules, and frozen in a tissue-freezing medium. Frozen sections were obtained by using the Cryostato (CM1900, Leica) and were then immunostained with mouse anti-*E. coli* O157 antiserum. The cytoskeleton was counterstained by Rhodamine-phalloidin. Results were observed by using fluorescent microscopy.

## Supporting Information

File S1 Ethical Inspection.
(PDF)
