# Supplementary material for: The N-Terminal Domain of EspF Induces Host Cell Apoptosis after Infection with Enterohaemorrhagic Escherichia coli O157:H7
Source: PLoS One. 2013 Jan 25;8(1):e55164. doi: 10.1371/journal.pone.0055164 (PMC3555930; doi:10.1371/journal.pone.0055164)
Supplement: File S1 — Ethical Inspection. (PDF) [file pone.0055164.s001.pdf]

南方医科大学实验动物伦理审查表

The Tab of Animal Experimental Ethical Inspection of Laboratory Animal Centre, Southern Medical University

编号(Number)：伦（审）2009-052

课题名称<br>(Experiment title)	肠出血型大肠埃希菌 O157:H7 分泌蛋白 EspF 功能研究<br>Study of the Effector EspF of EHEC O157:H7		
课题负责人<br>(Project director)	万成松<br>Chengsong. Wan	联系电话<br>(Tel.)	020-61648304
所在单位<br>(Name of organization)	南方医科大学<br>Southern Medical University	课题来源<br>(Project sources)	广东省科技计划项目<br>The Natural Science Foundation of Guangdong Province (No. 2010B031000005)
实验动物使用许可证号<br>(Number of the using of Laboratory Animal)	SCXK 粤 2006-0015		

项目意义及必要性 (Significant)

(包括选用该种动物和数量的依据，有无替代方法，实验是否符合“3R”原则等)

本项目从南方医科大学实验动物中心购买 30 只 4~5 周龄 BALB/c 小鼠，研究肠出血型大肠埃希菌 O157:H7 EspF 功能。虽然 EHEC O157:H7 能够感染 C3H/HeJ、C57BL/6 和 BALB/c 小鼠，但在接种 EHEC 时，腹腔注射丝裂霉素 C，并于饮水中加入链霉素，可提高 BALB/c 小鼠易感性。

In this study, thirty BALB/c mice (4-5 weeks old, 13.27 ± 1.27 g weight) are infected EHEC O157 WT and Δ*espF* mutant. In order to improve the mice's susceptibility, we choose BALB/c mice instead of C3H/HeJ, C57BL/6 and provide mitomycin C in the experiment.

动物处置方法 (Outline of experiments)

(包括手术类型、手术后的护理、麻醉、安乐死方法等。)

(experimental methods, observational index, executing animal method, et. al)

该研究本着实验动物福利和伦理的原则，本实验项目优化设计方案，严格计划动物需要数量。实验方法涉及各组小鼠灌胃前同时腹腔注射丝裂霉素 C 溶液，二次给药后，每日观察小鼠攻毒后的临床表现，检测小鼠粪便排菌情况，统计小鼠攻毒后存活情况。实验 20 天，麻醉小鼠，实施小鼠安乐死，并按无害化处理要求包装后再冻存，最后由学校专门机构统一收集并焚烧。

In this study, the 30 BALB/C mice are injected with mitomycin C (MMC; 2.5 mg/kg) and provided water containing nalidixic acid (50 μg/mL) before infection. After the secondary infection poison, the survival rate will be assessed daily for 20d. After the experiments, all mice will be sacrificed by mean of diethyl ether anesthesia and burned.

声明(Statement):

1、我保证本研究动物实验的人员接受了生物学、操作、动物护理方面的训练；一些必要的无菌手术方法和技术；限制动物使用或不良应激减少到最小的概念、可行性、研究方法或检测方法；镇痛药、镇定剂和麻醉剂的合理使用；出现问题时向有关部门报告的程序。

2、如该研究方案有重大变更，事前一定获得实验动物管理和伦理委员会的许可。

3、我将自觉遵守实验动物福利伦理原则，随时接受实验动物伦理委员会的监督与检查，如违反规定，自愿接受处罚。

(I will conscientiously abide by the ethical principles of animal welfare, accept the supervision and inspection of the committee at any time, and voluntarily accept the punishment if any infringement.)

声明人签字：万成松 万成松　　2009 年 4 月 13 日

伦理委员会审批意见(Attitude of the Animal Management and Ethics Committee):

经审查、符合动物福利与伦理原则[illegible]，同意实施。

主要审查人签字	袁进	南方医科大学实验动物伦理委员会<br>Laboratory Animal Ethics Committee of Southern Medical University<br>2009 年 4 月 20 日
批准人签字	顾为望	

注：1、审查表纸质版两份：一份存档、一份交实验人员；电子版一份，发送至实验动物中心邮箱：dwgy61648042@126.com；2、如实填写表中所有内容。
